# In Vivo Seeding of Amyloid-β Protein and Implications in Modeling Alzheimer’s Disease Pathology

**DOI:** 10.3390/biom15040571

**Published:** 2025-04-11

**Authors:** Qianmin Liu, Simin Song, Lu Liu, Wei Hong

**Affiliations:** 1 School of Biomedical Sciences, Hunan University, Changsha 410082, China; qm.liu@siat.ac.cn; 2 Shenzhen Key Laboratory of Neuroimmunomodulation for Neurological Diseases, Shenzhen-Hong Kong Institute of Brain Science, Shenzhen Institutes of Advanced Technology, Chinese Academy of Sciences, Shenzhen 518055, China; songsm@hku-szh.org; 3 Department of Chinese Medicine, The University of Hong Kong-Shenzhen Hospital (HKU-SZH), Shenzhen 518055, China

**Keywords:** amyloid β-protein, biological seeds, intracerebral inoculation, amyloidosis, tau pathology, cognitive decline

## Abstract

Alzheimer’s disease (AD) is a progressive neurodegenerative disorder characterized by extracellular plaques containing amyloid β-protein (Aβ) and intracellular neurofibrillary tangles formed by tau. Cerebral Aβ accumulation initiates a noxious cascade that leads to irreversible neuronal degeneration and memory impairment in older adults. Recent advances in Aβ seeding studies offer a promising avenue for exploring the mechanisms underlying amyloid deposition and the complex pathological features of AD. However, the extent to which inoculated Aβ seeds can induce reproducible and reliable pathological manifestations remains unclear due to significant variability across studies. In this review, we will discuss several factors that contribute to the induction or acceleration of amyloid deposition and consequent pathologies. Specifically, we focus on the diversity of host animals, sources and recipe of Aβ seeds, and inoculating strategies. By integrating these key aspects, this review aims to offer a comprehensive perspective on Aβ seeding in AD and provide guidance for modeling AD pathogenesis through the exogenous introduction of Aβ seeds.

## 1. Introduction

Alzheimer’s disease (AD) is a widespread neurodegenerative disorder that is primarily characterized by progressive memory loss, notable changes in personality, and eventual severe cognitive decline and dementia [1,2]. Epidemiological studies indicate that AD can be categorized into a sporadic form (accounting for 95%) and a familial form (accounting for 1–5%), the latter of which is linked to specific genetic mutations [2,3,4]. Compelling data from genetic and clinical studies have suggested an initiating role of the amyloid β-protein (Aβ) in AD pathogenesis. The formation of extracellular amyloid plaques, primarily composed of aggregated Aβ, occurs years before disease onset and eventually induces neurofibrillary tangles and synaptic loss [5]. The amyloid hypothesis proposes that the deposition of Aβ in the brain parenchyma triggers a cascade of neurotoxic events and leads to widespread neurodegeneration [5,6,7]. However, recent studies have shown that there is a more complex AD etiology in which the role of Aβ in neurodegeneration is linked to multifactorial aspects [8].

Given the complexity and multifaceted nature of AD, as well as the difficulty of obtaining human resources, there is an urgent need for well-characterized animal models in the exploration of disease mechanisms and the development of early diagnostic and therapeutic strategies before translating to humans [9,10,11]. To date, hundreds of AD animal models have been developed. However, very few adequately replicate the complex neuropathological and behavioral phenotypes observed in AD patients [12,13,14]. For instance, transgenic mice that overexpress mutant amyloid precursor protein (APP) and/or APP/Presenilin 1 (PS1) recapitulate AD-like pathological features such as Aβ accumulation and neuroinflammation but also have artificial phenotypes due to excessive production of mislocalized APP/PS1 and their fragments [12,15,16,17]. To address these issues, single *APP* knock-in (KI) mouse models harboring the Swedish and Beyreuther/Iberian mutations were developed with a genome editing approach [18]. These mice exhibit Aβ pathology and neuroinflammation in an age-dependent manner and require up to 18 months to gain apparent cognitive impairment [18]. While introducing the Arctic mutation effectively accelerates the onset of memory decline, this model is not suitable for investigating Aβ metabolism and clearance, as the mutated Aβ is resistant to proteolytic degradation [18,19].

**Figure 1:**
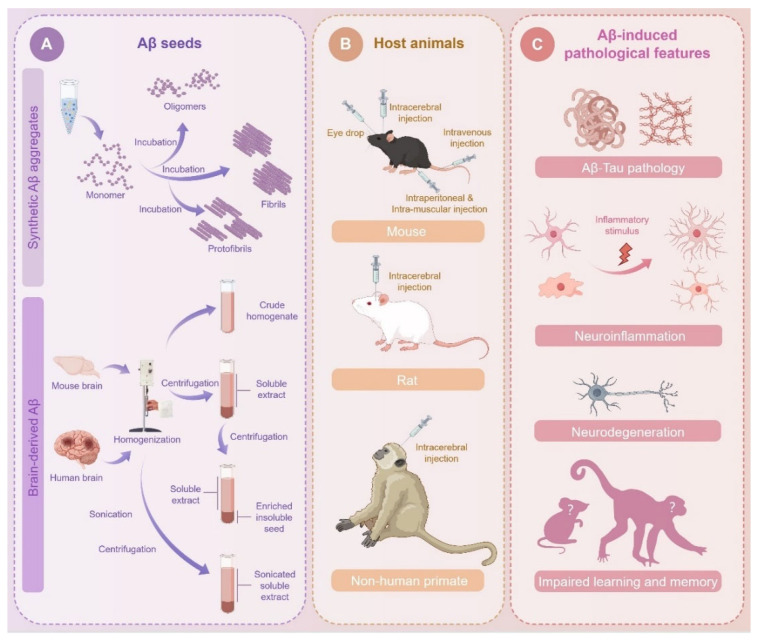
Variables in seeds, host animals, and injection approaches for Aβ seeding. (**A**) Different types of synthetic Aβ aggregates are prepared by incubating monomer under certain conditions. Biological Aβ seeds are prepared from AD patient or mouse brain tissues through a series of processing steps, including mechanical homogenization, centrifugation, and sonication. (**B**) Aβ seeds can be introduced through different routines, including intracerebral and peripheral injection. (**C**) Aβ injection induces multiple AD-like pathological features in host animals, such as Aβ pathology, tau pathology, neuroinflammation, neuronal degeneration, and impaired learning and memory behaviors.

Emerging data from animal and human studies suggest that Aβ pathology can be induced by the injection of pre-formed Aβ seeds, which can initiate a cascade of downstream events, including neuroinflammation and memory loss [20,21,22,23]. Taking advantage of the Aβ seeding phenomenon and animal models, significant efforts have been made to develop inducible AD models that may recapitulate key disease features. This review aims to provide a comprehensive summary of the literature on in vivo Aβ seeding activity and its consequent disease manifestations. We will discuss three major factors that may influence this process: the host animals, the types of Aβ seeds, and the injection approaches (Figure 1). We also discuss the advantages of modeling AD pathology using the Aβ seeding approach, as well as the challenges that require further investigation.

## 2. Rodents and Primates as Host Animals for Aβ Seeding

Exogenous seeding of Aβ pathology has been successfully achieved in various animal models, including rodents and non-human primates (NHPs) [20,21,24,25]. Commonly used rodents for seeding investigation include mice and rats that express multiple transgenes related to familial AD, such as mutant *APP* and *PS1* [26]. These animals produce high levels of humanized Aβ and can develop pathology relatively early in life, making them valuable for studying the mechanisms of Aβ propagation and seeding.

### 2.1. Genetic Modified Mouse Models

Compared to human Aβ, murine Aβ is less prone to forming aggregates due to differences in three amino acids at the N-terminal positions 5, 10, and 13 [27]. Murine Aβ is also difficult to seed on its own or to cross-seed in the presence of pre-formed human Aβ aggregates [27,28]. Thus, nearly all investigations on the spreading of amyloid pathology rely on genetically modified murine models that carry humanized Aβ [29]. Genetic studies on human early-onset familial AD have identified various pathogenic mutations in the genes encoding *APP*, as well as *PS1* and *PS2,* which influence the activity of β-secretase and γ-secretase during APP proteolytic processing [30]. The introduction of such mutations, along with the expression of human Aβ, can induce a range of AD-related phenotypes in mice [24,30,31].

The first generation of mice that recapitulate human AD pathology includes models that overexpress a single gene with one or more mutations, most commonly the human *APP* gene. The PDAPP line is the inaugural mouse model to mimic AD pathogenesis, which employs the platelet-derived growth factor (PDGF)-β promoter to drive the expression of the human *APP* gene carrying the Indiana mutation (V717F) [16,32,33]. The Tg2576 and APP23 mouse models overexpress the human *APP* gene with the Swedish mutation (KM670/671NL), leading to elevated levels of Aβ and age-related deposition of amyloid plaques [34,35,36,37]. TgCRND8 mice overexpress the human *APP* gene carrying both the Swedish mutation (KM670/671 NL) and the Indiana mutation (V717F) [38]. The introduction of highly pathogenic mutations in the *APP*, *PS1*, and *PS2* genes causes advanced amyloid plaque deposition in the mouse brain, which progressively becomes more pronounced with age [34,39].

The second generation of transgenic mice takes advantage of multiple genes and mutations, which may contribute to the accelerated development of AD pathology. APP/PS1 is a double transgenic mouse model that overexpresses a chimeric mouse/human *APP* (Mo/HuAPP695swe) and a mutant human *PS 1* (PS1-dE9) in neurons and provides an important tool for understanding the interaction between APP and PS1 in AD pathology during aging [40,41,42,43]. The 5×FAD mice overexpress human *APP* and *PS1* genes with five mutations: the Swedish (K670N/M671L), Florida (I716V), and London (V717I) mutations in *APP* and the M146L and L286V mutations in PS1. This mouse model recapitulates many AD-related phenotypes and exhibits relatively early and aggressive progression. It has been one of the most widely used models over the past two decades [44,45,46]. The 3×Tg mice carry three mutations associated with familial AD (APP Swedish, MAPT P301L, and PS1 M146V) and develop age-related, progressive amyloid deposits, as well as synaptic dysfunction and cognitive impairment. This mouse model also produces hyperphosphorylated tau with age and develops extensive tau tangles due to the overexpression of mutant human tau, making it an ideal model for investigating both Aβ and tau pathology [47].

With the rapid advances in gene editing technology, a series of novel gene KI and knock-out (KO) murine models have been developed in recent years to better mimic AD pathogenesis. These gene-edited mouse lines eliminate several artificial effects and abnormal phenotypes caused by the random insertion of *APP* and/or *PS1/2* genes, as well as the uncertain production of these proteins. The APP ^NL-F/NL-F^ mice express APP at wild-type levels but produce high levels of humanized Aβ due to two pathogenic mutations, the Swedish (K670N/M671L) and the Iberian (I716F) [18]. These mice recapitulate several AD-related pathologies, including increased hydrolysis of APP, cerebral amyloid deposition, synaptic loss, and microgliosis and astrocytosis, but generally lack tau protein-associated neurofibrillary tangles and cognitive dysfunction until very advanced ages [18,48]. To address these important issues, the same group developed a new mouse line that expresses human MAPT protein accompanied with elevated levels of human Aβ by crossing human *MAPT*-KI mice with *APP*-KI mice [49]. The double-KI mice display comparable levels of amyloid plaques, neuritic dystrophy, neuroinflammation, and memory deficits to *APP* KI mice but exhibit more pronounced tau phosphorylation. No evidence of tau pathology or neurodegeneration is observed before 24 months of age, unless accelerated by inoculating AD brain-derived tau seeds [50]. These studies demonstrate that the new generations of gene-edited mice exhibit only moderate progression of AD pathology and cognitive decline. They thus provide ideal models and a critical time window to monitor disease onset in the absence and presence of exogenous protein seeds and to dissect the role of the Aβ–tau axis in the etiology of AD.

### 2.2. Rat Models

Compared to mice, rats are closer to humans in terms of physiological structure and genetic characteristics. Their nervous system and cognitive functions are also significantly more complex than those of mice, making rats an important model for studying neurological diseases. The McGill-R Thy1-APP rat model, the most extensively studied APP transgenic rat strain, expresses human APP751 with the Swedish and Indiana mutations. It exhibits age-dependent accumulation of amyloid plaques, gliosis, loss of cholinergic synapses, and cognitive deficits [51,52]. The TgF344 rat model expresses human APP695 that carries the Swedish and PS1ΔE9 mutations, and develops region-wide amyloid accumulation [53]. Compared to McGill-R Thy1-APP rats, TgF344 rats exhibit significant tau phosphorylation and neurofibrillary tangles but lack synaptic loss and neurophysiological dysfunction [51,52,53]. Following the strategy used to develop *APP KI* mice, a research group has successfully developed an *APP KI* rat model that exhibits typical Aβ plaque deposition, microglial activation, gliosis, synaptic damage, and cognitive dysfunction. Most strikingly, these rats also develop AD-like phenotypes not commonly observed in other models, such as tau pathology, neuronal apoptosis, necrotic cell death, and cerebral atrophy [19]. However, the E22G mutation within the Aβ region in this model drives rapid formation of atypical Aβ conformations, restricting its relevance to sporadic AD.

Over the past few decades, a large number of genetically modified murine models have greatly enhanced our understanding of AD. However, they are still inadequate for fully reflecting the complexity and diversity of biological events in humans due to several differences between rodents and humans, such as brain structure and function, the aging process, and immune and metabolic systems. For instance, the prefrontal cortex of the human brain is crucial for maintaining cognitive functions, whereas this region is absent in the mouse brain [54,55]. Additionally, AD is a slow and progressive degenerative disease that may take ten to twenty years to develop detectable pathological changes and significant clinical symptoms. It is inevitable that the immune system and metabolic imbalances will affect disease onset during aging [56,57]. In the context of Aβ seeding, species-specific differences in immune responses and metabolic efficiency could also affect Aβ clearance, thereby shaping the development of AD pathology.

### 2.3. Non-Human Primates (NHPs)

In contrast to rodents, NHPs exhibit a high degree of similarity to humans in various biological characteristics, including genetic background, cognitive behavior, immune response, and anatomical structure. They possess advanced brain functions and neurological activities that are unparalleled in rodents, making them more suitable for studying human neuropsychiatric diseases and for drug discovery. Both naturally aging NHPs and artificially induced models have been reported in recent years. Apes, such as chimpanzees, gorillas, and orangutans, have Aβ sequences that are identical to those of humans [58]. These animals produce Aβ that gradually accumulates in the brain with age, eventually forming amyloid plaques and cerebrovascular amyloid lesions (cerebral amyloid angiopathy, CAA) in older individuals. However, NHPs tend to have more CAA and fewer cerebral plaques compared to humans [58,59,60]. Histological examination indicates that NHP plaques are mostly diffuse, while human plaques are more extensively compact [61].

Chimpanzees have been found to develop cortical tauopathies similar to those in humans, but their cognitive decline differs from the cognitive deficits observed in human AD patients [58,60,61,62]. These animals are suitable for studying human-like tau pathology to some extent but cannot be used as common research models due to their long lifespan, large size, and ethical constraints. In contrast, smaller NHPs, such as rhesus monkeys, baboons, and cynomolgus monkeys, are more convenient for use as model animals. Rhesus monkeys share structural homology with humans in their Aβ sequence, and aged individuals exhibit readily detectable Aβ plaques with distribution patterns similar to those observed in human AD patients [63,64,65,66]. The majority of Aβ plaques in the brains of rhesus monkeys are diffuse, with only about 20% being dense and associated with minor neuronal loss, suggesting a significant difference in how Aβ pathology manifests in NHPs compared to humans [67,68]. Despite the high degree of tau sequence homology between humans and rhesus monkeys, the latter develop very rare neurofibrillary tangles [67,69]. Nonetheless, aging rhesus macaques exhibit progressive cognitive decline, likely due to their close genetic similarity to humans [70,71,72]. In aged vervets, Aβ and tau burdens are associated with reduced brain volume and glucose metabolism, as well as with abnormalities of complex integrated behaviors such as gait speed. In addition, the observed Aβ plaque accumulation shares a similar distribution throughout the cerebral cortex as in humans with early AD neuropathologic changes [73]. While naturally aged NHPs can develop pathological changes and memory impairments resembling human AD to some extent, the variability in these manifestations among individuals and the long lifespan required to observe significant outcomes have substantially limited their use in the laboratory.

To accelerate AD-like progression in NHPs, recent research has focused on creating inducible models by introducing pathogenic protein seeds. For instance, inoculating Aβ oligomers into the brains of rhesus monkeys has been shown to impair synaptic integrity, induce neuroinflammation, and elevate AD biomarkers in the cerebrospinal fluid (CSF) to levels comparable to those observed in AD patients [74]. Rhesus monkeys injected with an adeno-associated virus (AAV) expressing a double tau mutation (P301L/S320F) exhibit misfolded tau propagation comparable to that observed in humans. This propagation is accompanied by a robust neuroinflammatory response and elevated biomarkers of inflammation and neuronal loss in CSF and plasma [75]. A recent study reported the generation of transgenic cynomolgus monkey models expressing tau (P301L) through lentiviral infection of monkey embryos and an inducible model that adult monkeys receive AAV-delivered tau (P301L). Both models develop severe tauopathy, leading to enhanced generation of Aβ oligomers in the spinal cord and resulting in age-dependent neurodegenerative lesions, abnormal glucose metabolism, and motor dysfunction [76]. These studies underscore the potential of developing more advanced NHP models that closely mimic human AD pathology by exogenously introducing pathogenic protein seeds, either through direct cerebral injection or a viral delivery strategy.

## 3. Resources of Aβ Seeds

The propagation of AD pathology and the subsequent molecular and cellular events are significantly influenced by the diversity of inoculated Aβ seeds, including their sources, species, and preparation methods [77]. Aβ seeds can be categorized into synthetic Aβ aggregates, brain homogenates, and extracts from transgenic mouse brains or human brain tissue [78]. Understanding the impact of different Aβ seeds is essential for accurately modeling AD pathology and evaluating potential therapeutic strategies.

### 3.1. Synthetic Aβ Aggregates

Over the past three decades, chemically synthesized Aβ peptides have been extensively utilized to study AD pathology. When these synthetic Aβ aggregates are injected into the brains of mice or NHPs, they can induce advanced amyloid deposition and various behavioral changes [75,77,79,80,81,82,83,84,85,86,87] (Table 1). Recent studies have shown that the human brain contains highly diverse Aβ primary structures encompassing a large number of N- and C-termini [88,89]. Synthetic Aβ40 and Aβ42 aggregates are the most widely used seeds and have been shown to efficiently induce amyloid pathology and accelerate disease progression in transgenic AD mouse models [79,90,91]. Aβ aggregates, despite originating from the same primary peptide, can adopt various conformations that differ in their toxicity mechanisms and pathological effects [92]. In the brain, certain Aβ strains exhibit a preference for incorporating specific Aβ isoforms into their aggregates. This strain-specific incorporation suggests that the seeding activity and the pathological changes induced may depend on both the Aβ peptides present in the host animal and the inoculated seeds [93,94].

In most genetic-modified AD models, the overexpression or more efficient cleavage of APP leads to elevated production of Aβ peptides, with Aβ42 levels significantly surpassing those of Aβ40. The aggregation propensity and toxicity of Aβ peptides are significantly influenced by their primary sequences and interplays. Aβ40 and Aβ42 interact strongly at the level of primary nucleation but self-assemble into separate homomolecular structures during fibril formation from mixed solutions of both peptides [95]. An early study has shown that Aβ40 monomers specifically require Aβ40 oligomers to induce growth of mature fibrils, whereas Aβ42 monomers are less selective and are stimulated by all types of seeds [96]; others report that Aβ40 aggregation can be accelerated by Aβ42 monomers but not Aβ42 fibrils by promoting primary nucleation through interaction between the different monomeric species in solution [95,97]. Controversially, other studies demonstrate that the fibrillization of the Aβ40 monomer can be accelerated by Aβ42 fibrils or high-molecular-weight oligomers by the association of Aβ40 monomer with the ends of Aβ42 fibrils and vice versa [98,99,100]. Monomeric Aβ40 alters the kinetic stability, solubility, and morphological properties of Aβ42 aggregates and prevents their conversion into mature fibrils [97,99]. In fact, rather than the morphology of the amyloid fibrils, the Aβ42:Aβ40 ratio modulates the aggregation behavior of each species [96]. A change in the Aβ42:Aβ40 ratio greatly induces the different structural types of aggregates such as different-sized oligomers or fibrils with their different morphologies and flexibilities [96,101]. These studies collectively demonstrate that cross-seeding between Aβ40 and Aβ42 results in heterogeneous aggregates, with structural outcomes dictated by seed type and concentration. Importantly, the cross-seeding behaviors of distinct Aβ species cannot be reduced to a simplistic interpretation where one species directly induces or delays the other’s aggregation.

Several studies have shown that AD patients have significantly increased levels of variable truncated Aβ peptides in the brain, which may be related to Aβ aggregation and toxicity. These truncated forms, such as N-terminally truncated peptides AβpE3–x and Aβ4–x, have been detected in high abundance in brain tissue and CSF from both sporadic and familial AD patients [88,89]. Other studies have shown that the middle region fragment Aβ25–35 is capable of inducing neuronal apoptosis, causing morphological changes and inflammatory responses in microglia, and eventually leading to memory deficits of mice and rats [102,103,104,105,106]. These findings suggest that truncated Aβ peptides may contribute to the progression of AD by modulating the aggregation of full-length Aβ, thereby affecting the formation of amyloid plaques and neurodegenerative processes.

To prepare Aβ seeds for inoculation, chemically synthesized Aβ peptides have to undergo a complex and cautious procedure that ensures reproducible generation of aggregates. Typically, Aβ monomer is incubated at a certain temperature for 3 days to a week to promote aggregation, and the resulting Aβ aggregates are snap-frozen and stored at −80 °C for subsequent injections [86,91,107,108]. The advantage of using highly pure synthetic Aβ aggregates as seeds is that the induced pathological changes are specifically attributed to Aβ itself. This allows for a more controlled and direct investigation of Aβ’s role in the development of amyloid pathology, without interference from other potential factors or contaminants. A standardized protocol enables the preparation of large-scale Aβ aggregates using the same batch of peptide stock, ensuring the reproducibility of both the inocula and their effects on disease progression. This consistency is crucial for comparing results across different studies and laboratories. It is also possible to investigate the seeding activity of a specific strain or species of Aβ, as well as the cross-seeding between different types of Aβ or between Aβ and other co-factors.

One limitation of using synthetic Aβ aggregates as seeds is the potential of structural differences between batches or vendors, which could affect the consistency and reproducibility of seeded pathology. In future studies, biophysical characterizations of synthetic Aβ seeds—using techniques such as circular dichroism (CD), atomic force microscopy (AFM), or size-exclusion chromatography (SEC)—may help standardize the properties of seed preparations and bioactivity. A second limitation is that synthetic Aβ may lack certain post-translational modifications as observed in humans. For example, isoAsp7-Aβ has been identified as a major Aβ variant in AD patients’ brains [109,110]. Repetitive intravenous administration of synthetic isoAsp7-Aβ(1–42) robustly accelerates formation of classic dense-core amyloid plaques in the brain of transgenic mice [111]. However, most studies have not incorporated such modifications into the seeds, and thus their ability to replicate key pathogenic properties observed in native Aβ aggregates remains uncertain.

### 3.2. Biological Aβ Seeds Derived from Mouse Brain

Unlike synthetic Aβ aggregates, brain-derived seeds may retain post-translational modifications, structural complexity, and biological cofactors, making them more physiologically relevant for investigating the seeding activity of Aβ. Aβ seeds derived from the brains of transgenic mice are capable of inducing amyloid deposition and tau pathology, exhibiting pathological characteristics similar to those observed in AD patients [5,22,77,112,113,114,115,116,117,118,119,120,121,122,123,124] (Table 2). For example, transgenic mouse models such as APP23, APP/PS1, 5×FAD, Tg2576, and TgCRND8 are often used as donors of Aβ seeds because of the high load of Aβ in their brains and their ability to cause cognitive dysfunction [5,77,113,114,115,116,117,118,119,120,122,123] (Table 2). When Aβ seeds were inoculated into an APP-deficient mouse model for six months and the latter was used in a secondary dissemination, β-amyloidosis was still induced in the APP transgenic mice. This suggests that Aβ seeds can persist in the brain for extended periods and, when host Aβ is present, resume their dissemination and pathogenic activity [123]. These mouse models provide a reliable source of Aβ seeds for studying the complex propagation of amyloid pathology and subsequent disease manifestations.

However, the pathological features of widely used animal models exhibit significant heterogeneity in amyloid plaque morphology and biochemical composition. Donor animal brains may display a spectrum of Aβ deposits, including diffuse plaques, dense-core plaques, and vascular amyloid (e.g., CAA), which subsequently induce variable pathological phenotypes in host animal brains [5,22,77,112,114,121,125,126,127]. For instance, injection of Aβ seeds from APP23 mice into APP/PS1 mice leads to the appearance of diffuse filamentous Aβ, as well as dense plaques, which contrasts with the typical pathology observed in the APP/PS1 mice [77,112]. Thus, there is a strain-like behavior of Aβ seeds that gives rise to different pathological morphologies of cerebral amyloid deposition.

Human Aβs vary in primary structures (e.g., Aβ40, Aβ42, Aβ43) and post-translational modifications (e.g., pyroglutamate-modified Aβ, covalently cross-linked Aβ dimer), which are thought to play different roles in plaque formation. The variable Aβ peptides have their distinct aggregation kinetics and susceptibility to seeds. In certain transgenic mouse models such as APP/PS1, Aβ seeds are predominantly composed of Aβ42, which has strong propensity for aggregation and readily forms oligomers and fibrils [77,79,128,129]. Aβ42-rich seeds are more likely to induce the formation of diffuse plaques, which are characterized by their loosely structured morphology and extensive distribution [130,131]. In contrast, Aβ40 exhibits greater solubility in perivascular fluids, facilitating its accumulation within vascular walls [127]. When brain-derived Aβ seeds contain a higher proportion of Aβ40, the pathological manifestations in injected animal brains are more likely to favor vascular deposition, resulting in increased CAA and associated vascular dysfunction [132]. These findings indicate that the ratio of Aβ40 to Aβ42 varies across different mouse Aβ seeds, in which Aβ42 plays a more prominent role in plaque formation, while Aβ40 is more closely associated with vascular amyloid pathology [112,129].

In genetically modified animal models, the artificially introduced mutations in APP and/or PSEN1/2, or risk alleles like APOE4, are all contributing factors to Aβ production, aggregation, and clearance. APP23 and Tg2576 mice carry the APP Swedish mutation (K670N/M671L), which primarily enhances the cleavage efficiency of β-secretase (BACE) on APP, leading to age-related increase in total Aβ production and the Aβ40/42 ratio [36,37,133,134,135]. Other widely used animal models, such as APP/PS1, 5×FAD, 3×Tg, and *APP^NL-F/NL-F^* mice, harbor additional mutations (e.g., PS1 mutations or γ-secretase cleavage site mutations in APP) which significantly promote the generation of Aβ42 and increase the Aβ42/40 ratio [18,43,45,46,47,136,137,138]. The variability in Aβ profiles across different animal models, resulting from genetic modifications, plays a crucial role in both the activity of Aβ seeds and the resulting pathological manifestations when these models are used as hosts.

In addition to directly influencing the Aβ profile by genetic modifications, other genetic factors can contribute to inflammation, vascular dysfunction, and metabolic disorders, which may drive atypical amyloid deposition patterns. For instance, ApoE4 stabilizes soluble, cytotoxic, oligomeric Aβ and enhances fibrillogenesis and has been shown to accelerate early seeding of amyloid pathology by perturbing Aβ clearance and enhancing Aβ aggregation [139,140]. Thus, a consensus is that inoculating Aβ seeds from donor brains into animal models may yield variable results, and such pathological diversity underscores the importance of selecting appropriate donor Aβ seeds and host animals for a specific purpose.

### 3.3. Biological Aβ Seeds Derived from Human Brain

Compared to mouse Aβ, Aβ seeds derived from the brains of AD patients are more physiologically relevant and have been shown to be transmissible through experimental inoculation and certain medical or surgical procedures. Iatrogenic early-onset CAA has recently been identified in patients with a history of traumatic brain injury or other cerebral and extracerebral lesions requiring neurosurgery or other medical procedures, such as intravascular embolization with cadaveric dura mater extracts. In these patients, Aβ seed transmission was found to occur through exposure to cadaveric dura mater or contaminated neurosurgical instruments, often many years before the first intracerebral hemorrhage (ICH) event [141]. Human transmission of Aβ pathology and CAA has been reported in relatively young adults who died of iatrogenic Creutzfeldt–Jakob disease (CJD) following childhood treatment with cadaver-derived pituitary growth hormone contaminated with both CJD prions and Aβ seeds [23,142,143,144]. These clinical findings demonstrate that Aβ derived from AD patients can propagate between individuals through iatrogenic routes, highlighting the potent transmissibility of Aβ seeds in vivo.

Animal studies have provided strong experimental evidence supporting the prion-like propagation capacity of Aβ seeds derived from AD patients [21,22,77,93,125,145,146,147,148,149,150,151,152] (Table 3). Injection of Aβ seeds extracted from AD patient brains into mice has been shown to induce amyloid plaque formation and CAA in the recipient animals [22,23,93]. These findings collectively highlight the potent ability of human-derived Aβ seeds to drive amyloid pathology and offer critical insights into the mechanisms underlying Aβ propagation. However, human Aβ can exist in numerous forms, differing in primary structure, conformation, size, and disease-related bioactivity [88,153]. Therefore, the spreading capacity of human Aβ seeds and the resulting pathological changes in host brains may vary significantly upon inoculation. Furthermore, the genetic background, clinical history, and disease stage of brain donors may also influence the amyloid-inducing activity of Aβ seeds.

Aβ derived from human brain tissue exists in multiple truncated forms, assembling into a wide range of structures, from dimers to high-ordered soluble and insoluble aggregates [88,154,155,156,157]. For example, Aβ40 and Aβ42 monomers can adopt distinct conformations, which may influence their aggregation behavior in the brains of AD patients [154,156,157]. The complex aggregation process of Aβ results in polymorphic aggregates with diverse structures and varying toxic effects [88,158,159]. Notably, the toxic effects induced by Aβ derived from AD brains are often hundreds to thousands of times more potent than those caused by synthetic Aβ aggregates [77]. Previous studies have shown that Aβ derived from the brains of AD patients can inhibit long-term potentiation, cause synaptic loss, induce neuronal excitotoxicity, and promote hyperphosphorylation of tau proteins [88,153,160]. Despite these findings, whether the neurotoxic effects and cerebral seeding activity of Aβ share a direct or mechanistic link remains unclear.

Several studies have demonstrated that while brain tissue from AD patients contains abundant diffuse and dense Aβ deposits, mice inoculated with human AD brain homogenates develop predominantly diffuse Aβ lesions and relatively few dense-core deposits [121,148,152,161,162]. Hippocampal injection of Aβ-rich brain homogenates from AD patients into APP/PS1 mice induces rapid Aβ deposition, neuronal damage, and intracellular Aβ aggregation in synaptic regions [149]. Similarly, injecting AD brain homogenates into the lateral ventricles of rats induces hippocampal microglial activation, triggers inflammatory responses, and reduces synaptic protein expression and brain volume [21]. In addition to rodent studies, several reports have described the induction of Aβ and tau pathology and cognitive decline in NHPs following inoculation of human brain-derived protein seeds [81,87,150,151,163,164]. These findings underscore the value of using human brain-derived Aβ seeds to inoculate mice and NHPs, providing biologically relevant models to study AD pathogenesis.

Islet amyloid polypeptide (IAPP) and pro-islet amyloid polypeptide (proIAPP) have been detected in cerebral and vascular Aβ deposits of AD patients, while Aβ reactivity is not present in islet amyloid extracts from patients with type 2 diabetes. Intravenous injection of preformed fibrils of synthetic IAPP, proIAPP, or Aβ in transgenic mice expressing human IAPP can act as a seed for IAPP amyloid in the islets of Langerhans. The heterologous seeding between IAPP and Aβ shown here may represent a molecular link between type 2 diabetes and AD [165]. Moreover, Aβ and α-synuclein have been shown to co-aggregate and form complexes in patient brains and transgenic models, providing clear evidence for their direct interaction [166]. Different structural forms of α-synuclein exert different effects on Aβ aggregation. Monomeric α-synuclein blocks the autocatalytic proliferation of Aβ42 fibrils, whereas fibrillar α-synuclein catalyzes the heterogeneous nucleation of Aβ42 aggregates [167]. Co-oligomer formation of α-synuclein and Aβ is generally more favorable than self-oligomer formation of α-synuclein at equilibrium [168]. These studies suggest that diabetes and other neurodegenerative disease may be risk factors for AD and that the cross-seeding of Aβ and other amyloidogenic proteins could play a critical role in AD pathology. Future studies investigating Aβ seeding and AD modeling should incorporate these cofactors to improve the relevance of disease models.

### 3.4. Methodologies for the Preparation of Brain-Derived Aβ Seeds

The methods for the preparation of biological Aβ seeds from animal or human brains vary widely between laboratories and in publications from the same group. In most cases, cortical tissue was mechanically homogenized in an aqueous buffer, diluted, and directly used as Aβ seeds. Only in a few cases were brain homogenates centrifuged, and the supernatant was used as the seeds [23,120,121]. Specifically, brain tissue was homogenized at 10% (*w*/*v*) in phosphate-buffered saline (PBS), then vortexed and sonicated three times for 5 s, and centrifuged at 3000× *g* for 5 min, and the supernatant was used as the Aβ seeds [77,79,112,114,123,125,148,152,161,169]. Alternatively, brain extracts can be further fractionated by ultracentrifugation to assess the seeding activity of both soluble and insoluble Aβ species. This distinction is important, as the morphological appearance of the induced Aβ deposits differs between the two types of seeds. Aβ deposits induced by insoluble seeds tend to be larger, often forming congophilic aggregates that appear nonuniformly distributed throughout the injected area. In contrast, soluble Aβ species from brain extracts typically induce smaller, Congo-red-negative patches of Aβ aggregates, which are more evenly distributed throughout the injected region [5].

The methodologies used to isolate biological Aβ seeds from brain tissue have several limitations. First, direct mechanical homogenization of brain tissue may disrupt the structural integrity of Aβ aggregates, potentially altering their pathological propagation effects [153,159,170]. Second, ultrasonic fragmentation may cause protein denaturation or disrupt the interactions between Aβ and other proteins or lipids, which might be critical in driving amyloid propagation [171]. Lastly, insufficient centrifugation of brain homogenates may fail to completely remove tissue or cell debris, potentially leading to artifacts in the induced pathology [120]. To address this issue, we previously developed a new gentle extraction protocol by soaking minced AD brain tissue in aqueous buffer. Next sequential centrifugation removes cellular debris and then large macromolecular assemblies. The resultant supernatant contains readily diffusible Aβ species, which make up a minority of total Aβ in AD brains but account for essentially all bioactivity present in extracts of homogenized brain [153,172]. Nonetheless, the variability in Aβ states in brain tissue and their neurotoxic effects support the notion that human Aβ is highly heterogeneous [172,173].

The amyloid-inducing activity of Aβ seeds can also be modified before injection. For example, pre-treating mouse brain-derived Aβ seeds with formic acid completely abolishes their amyloid-inducing activity, while heat treatment partially reduces but does not eliminate the seeding activity of mouse brain extracts [77]. Formaldehyde fixation alters the morphology of Aβ plaques in the donor brain and affects the subsequent seeding activity of the extracted seeds. Brain homogenates prepared from unfixed frozen tissue of APP23 mouse results in diffuse plaques in the host brain, whereas samples from formaldehyde-fixed tissue produce more punctate deposits [119]. Extended sonication of mouse brain extracts enhances the in vivo seeding activity of Aβ, possibly by generating smaller, fragmented aggregates from larger insoluble assemblies.

Collectively, prior studies have demonstrated that biological Aβ seeds isolated from mouse or human brains are a critical tool for investigating the molecular mechanisms underlying Aβ propagation in AD. Although Aβ seeds vary in their original amyloid morphology, molecular composition, and relative abundance, their ability to induce β-amyloidosis in host animals has provided valuable insights into the molecular pathology of AD. However, significant challenges remain in standardizing seed preparation methodologies, understanding strain-specific differences in Aβ seeding, and translating these pathological findings into accurate AD models. Conformation- and/or site-specific antibodies, such as A11, may be useful in characterizing the structural features and bioactivities of synthetic or brain-derived Aβ seeds.

### 3.5. Structural Features of Different Types of Aβ Seeds Acquired from in Vitro Synthesis, Mouse Brain, and Human Brain

Variations in Aβ fibril structure in vivo may correlate with differences in AD phenotype, in analogy to distinct prion strains that are associated with different clinical and pathological phenotypes. There have been many attempts to obtain high-resolution structural information about Aβ fibrils. Most of previously reported Aβ fibril structures are in-register, parallel, cross-β-sheets that mostly consist of two protofilaments twisted around each other. An atomic resolution structure of Aβ42 fibrils identified by magic angle spinning (MAS) nuclear magnetic resonance (NMR) spectroscopy reveals parallel, in-register cross-β-sheets, with the fibril core consisting of a dimer of Aβ42 molecules, each containing four β-strands in an S-shaped amyloid fold and arranged in a manner that generates two hydrophobic cores that are capped at the end of the chain by a salt bridge [174]. Another study combining solid-state NMR spectroscopy and mass-per-length measurements from EM shows that the 3D structure of Aβ42 fibrils is composed of two molecules per fibril layer, with residues 15–42 forming a double-horseshoe-like cross-β-sheet entity with maximally buried hydrophobic side chains. Residues 1–14 are partially ordered and in a β-strand conformation but do not display unambiguous distance restraints to the remainder of the core structure [175]. As determined by cryo-electron microscopy (cryoEM) complemented by solid-state NMR, the structure of the Aβ42 fibril is composed of two intertwined protofilaments in which the N terminus serves as part of the cross-β structure, resulting in an overall “LS”-shaped topology of individual subunits [176]. Another study has reported that the Aβ42 fibril displays triple parallel β-sheet segments that differ from others. The Aβ40 fibril is incompatible with the triple-β-motif, because seeding with Aβ42 fibrils does not promote conversion of monomeric Aβ40 into fibrils via cross-replication [177]. The spherical amyloid assembly of the synthetic Aβ42 structure involves a β-loop-β motif, which significantly differed from the triple-β motif observed for the Aβ42 fibril [178]. Solid-state NMR analyses of soluble Aβ oligomers prepared from recombinant Aβ42 revealed a mixed parallel and antiparallel β-sheet structure that is different from fibrils which contain only parallel β-sheets [179].

Solid-state NMR analysis of tissue from two AD patients with distinct clinical histories identified a single predominant Aβ40 fibril structure in each patient, but different from one another and distinguishable from fibrils produced in vitro [156]. The predominant molecular structure in brain-seeded fibrils differs from the structures of purely synthetic Aβ40 fibrils [180]. Solid-state NMR measurements of Aβ40 and Aβ42 fibrils prepared by seeded growth from extracts of AD brain cortex demonstrate the existence of a specific predominant Aβ40 fibril structure in different AD clinical subtypes. There is also a qualitative difference between Aβ40 and Aβ42 aggregates in the brain tissue of patients with AD [154]. Further study involved a high-resolution cryoEM density map that showed that these fibrils have a four-layered cross-β structure, with twofold screw symmetry about the fibril growth direction and with fully extended conformations for Aβ40 molecules in the inner layers [181]. Structural analysis with cryoEM showed that brain-derived Aβ amyloid fibrils are right-hand twisted and their peptide fold differs sharply from previously analyzed Aβ fibrils that were formed in vitro [182,183]. CryoEM structures of Aβ42 filaments from human brain reveal two structurally related S-shaped protofilament folds that give rise to two types of filaments. Type I filaments are found mostly in sporadic AD and are made of two identical S-shaped protofilaments embracing each other with extended arms, and each protofilament comprises five β-strands. Type II filaments are mostly found in familial AD and APP^NL-F^ mice and contain an ordered core that extends from 12 to 42 and comprises four β-strands [183].

Most Aβ filaments in AD patients with the Arctic mutation (Aβ E22G) consist of two pairs of non-identical protofilaments that comprise residues V12-V40 or E11-G37, whereas most filaments in APP^NL-G-F^ mice are made of two identical mutant protofilaments that extend from D1 to G37 [184]. Parenchymal deposits of Aβ42 and blood vessel deposits of Aβ40 have distinct structures, supporting the view that AD and CAA are different Aβ proteinopathies [185]. Three types of Aβ40 filaments have been identified from the leptomeninges of individuals with AD and CAA, and each comprises one, two, or three protofilament pairs [185]. A recent study reports two types of Aβ40 filaments in adult individuals with Down syndrome (DS) that differ from those in sporadic AD and two types of Aβ42 filaments identical to those found in sporadic and familial AD [186]. The presence of structural variations among Aβ aggregates in the human and transgenic mouse brains, as well as environmental effects on the propagation of specific structures, should not be overlooked in the study of Aβ seeding. A central goal for future work is to determine whether structurally distinct Aβ aggregates can consistently seed different patterns of Aβ pathology in suitable animal models.

## 4. Experimental Strategies for Aβ Inoculation

The regional specificity of Aβ production and accumulation in each animal model or human donor may play a pivotal role in the development of AD pathology and associated cognitive impairment. For example, in 5×FAD mice, the deposition of Aβ plaques is positively associated with several brain regions, including the prefrontal cortex, somatosensory cortex, medial amygdala, thalamus, and hippocampus [187]. In humans, neuropathological studies indicate a spatiotemporal evolution of Aβ accumulation, beginning in cerebral regions with neuronal populations characterized by high metabolic and bioenergetic activity (such as the association cortices). This accumulation then spreads from the neocortex to the allocortex, brainstem, and eventually the cerebellum [188,189]. The varying vulnerability of distinct brain regions to Aβ accumulation is closely related to the specific molecular properties of the affected neural systems [190,191]. The injection site of Aβ seeds may play a crucial role in inducing amyloid deposition and could significantly influence subsequent pathological events.

### 4.1. Intracerebral Injection

In prior studies, Aβ seeds are typically administered via intracranial injection into the hippocampus and cortex—regions highly susceptible to Aβ deposition in AD [25,75,77,86,114,118,122,125,145,146,149,151,162,192,193]. Transgenic animals injected with synthetic Aβ aggregates or seed-containing brain extracts into the hippocampus exhibit early Aβ deposition, tau pathology, and memory impairment [5,77,118,145,146,150,193]. For example, both Tg2576 mice and APP21 rats developed senile plaques and CAA in the injected brain region following hippocampal injection of human AD brain extracts [22,162]. APP^NL-F/NL-F^ mice injected with human and mouse brain extracts in the parietal lobe exhibit plaque deposition and CAA [194]. Although all injected brain regions (i.e., the olfactory bulb, parietal cortex, entorhinal cortex, striatum, and hippocampus) have the potential for the induction of Aβ deposition, there is significant difference between the amount and type of amyloid induced across these areas. For example, in the entorhinal cortex and hippocampus, amyloid induction was robust and predominantly congophilic, whereas in the striatum, far less amyloid was induced, and the deposits were primarily diffuse [113].

The induction of Aβ deposits is most pronounced in the vicinity of the injection site, yet it was also detectable throughout the entire injected brain area [113,125]. This spread likely involves both passive diffusion of soluble Aβ and active transport along axonal and synaptic connections. Other studies have shown that lateral ventricle injection of Aβ may distribute the seeds throughout the brain, thereby accelerating Aβ propagation [21,74,83]. For instance, rhesus monkeys injected with synthetic Aβ into the lateral ventricle develop age-related Aβ and tau pathology, accompanied by declines in learning and memory abilities [74]. Collectively, these studies suggest that the brain regions in which seeds are inoculated may affect the type of Aβ pathology that ultimately develops.

### 4.2. Peripheral Injection

It has been shown that an infectious prion particle can infect the host by various peripheral application routes. Cerebral Aβ amyloidosis can also be induced by peripheral injection of seeding-competent aggregates [114]. Intraperitoneally inoculated Aβ-containing brain extracts can induce the accumulation of cerebral Aβ in host animals at multiple loci, most efficiently in regions with high availability of soluble Aβ [114,115]. A recent study demonstrated that cerebral accumulation of Aβ can be accelerated after exposing mouse models of AD to Aβ seeds by different peripheral routes of administration, including intra-peritoneal and intra-muscular. Drops of brain homogenate laden with Aβ seeds in the eyes can efficiently induce amyloid deposition, while oral administration of large quantities of brain extracts does not have any effect [22]. Thus, cerebral β-amyloidosis can be seeded by homologous protein aggregates delivered into the peritoneal cavity following a similar pattern as prion disease, but the efficiency of peripheral administration routes may require more time and are less efficient than that of intracerebral injection [113,114]. The mechanism behind the peripheral induction of cerebral amyloidosis is not yet fully understood, but it is possible that certain Aβ seeds may either cross the blood–brain barrier or activate systemic pathways that promote cerebral amyloidosis.

## 5. Conclusions and Future Directions

Despite recent progress in drug discoveries, there are still needs in the field to develop more diverse animal models for the goals of studying heterogeneous mechanisms and searching for new targets of AD. Significant evidence has shown that inoculation of brain homogenate extracts containing certain Aβ seeds can induce AD-like pathology in transgenic murine models of amyloidosis or in NHPs [69,86,150,195,196]. Thus, modeling disease progression by Aβ seeding strategy may open a new path for specific research questions to be addressed in the AD field.

The advantage of the seeding strategy is that cerebral Aβ injection often results in rapid amyloid deposition in host animals, and the procedure can be easily controlled and manipulated. For instance, by injecting Aβ seeds into specific brain regions like the hippocampus or cortex, researchers can precisely investigate regional pathological changes and their projections to other brain areas [22]. Additionally, mouse models of amyloidosis typically do not develop robust tau pathology on their own but can be induced following intracerebral injection of Aβ-containing brain extracts. This approach provides a valuable tool for studying the synergistic effects of Aβ and tau in AD [197,198,199].

One major challenge in the field is the variability in the host animals used for Aβ seeding across different studies and laboratories. The vast majority of studies were performed on transgenic mouse or rat models that overexpress human *APP* and/or *PS1* genes with genetic mutations associated with familial AD. While these models allow for efficient and rapid detection of pathological changes resembling familial AD, the drawbacks of aggressive gene manipulation cannot be overlooked. The most recently developed gene-edited animals may avoid some or all of these side effects. In the future, it will be essential to use newer generations of mouse models through KI/KO or CRISPR gene editing technologies or NHPs, which may more accurately model AD progression and avoid potential side effects caused by gene overexpression.

It remains unclear to what extent these animal models can capture the complexity and diversity of biological events observed in humans, given the significant differences between rodents and humans in brain structure, function, aging processes, and immune and metabolic systems. For example, the human prefrontal cortex plays a crucial role in maintaining cognitive functions, whereas this region is largely absent in the mouse brain. In addition, AD is a slow and progressive degenerative disorder that can take decades to develop significant pathological changes and clinical symptoms. A critical limitation of gene-edited murine models is their tendency to develop AD-like pathology at a very young age, raising an important question of whether aging should be more considered when modeling AD pathogenesis in these systems.

Another challenge is the variability in outcomes resulting from the diverse sources of Aβ seeds. Due to the limited availability of human brain tissue, many studies have relied on preformed fibrils generated from synthetic peptides that may not fully replicate the complex and heterogeneous nature of Aβ aggregates found in human brains. In contrast, brain-derived seeds may retain post-translational modifications, structural complexity, and associated biological cofactors, making them more physiologically relevant for investigating the prion-like propagation of Aβ pathology. However, a critical challenge in using brain-derived Aβ seeds is the potential modulation of their seeding potency by donor-specific factors, particularly genetic polymorphisms and disease stage. To develop a more accurate and consistent model, it is essential to better characterize the propensities of different host animals to develop Aβ deposition and carefully select the most suitable source of Aβ seeds.

The third challenge is the inconsistency in the methods used to prepare biological Aβ seeds from animal or human brains across different laboratories and publications. To better understand the mechanisms underlying seed-induced Aβ pathology and establish reliable models of AD pathogenesis, it is essential to systematically evaluate and standardize seed preparation and administration protocols. This includes, but is not limited to, brain tissue handling, seed isolation and purification, biochemical characterization, and the selection of appropriate inoculation sites. Standardizing these methodologies will help ensure consistency and reproducibility in experimental outcomes, ultimately advancing our understanding of Aβ propagation and AD progression.

## Figures and Tables

**Table 1:** Seeding of synthetic Aβ aggregates in different host animals.

Host Animal	Aβ Seeds	Injection Site	Induced Pathological Features	Reference
APP23 mice	AβM1–42 fibrils AβM1–40 fibrils	Right cerebral hemisphere	Advanced Aβ plaques Glial activation Mild cognitive deficits	[82]
	Aβ1–42 oligomers Aβ1–40 oligomers	Cortex or striatum		[79,91]
	Aβ1–40 fibrils Aβ1–42 fibrils	Hippocampus	No detectable Aβ deposition	[77]
Wistar rats	Aβ1–42 aggregates	Cerebroventricular	Memory impairment Neuronal death	[83]
Wistar rats (*Rattus norvegicus*)	Aβ1–42 oligomers	Lateral ventricle	No fibrillar amyloid deposits Tau phosphorylation	[85]
Cynomolgus macaques (*Maccaca fascicularis*)	Aβ1–42 oligomers	Lateral ventricle	No fibrillar amyloid deposits Increased Tau phosphorylation Increased neurofibrillary tangles Astrocyte and microglia activation Apparent synapse loss	[85]
Cynomolgus monkeys	Aβ1–42 oligomers	Brain parenchyma	Increased amyloid plaques Increased neurofibrillary tangles Profound neuroinflammation Degenerative neurons and synapses	[86]
Marmoset monkeys	Freshly dissolved Aβ1–40 or Aβ1–42	Multiple sites *	No induced amyloid	[87]
Rhesus monkeys	Aβ1–42 oligomers	Cerebroventricular	Reduced spine density Microglia activation Induced neuroinflammation Increased CSF markers	[75]

* Caudate and accumbens in one hemisphere, hippocampus and amygdala in the opposite hemisphere, parietal cortex in both hemispheres.

**Table 2:** Seeding of mouse brain-derived Aβ seeds in different host animals.

Host Animal	Sources of Aβ Seeds	Injection Site	Induced Pathological Features	Reference
APP23 mice	22–28 months APP23 mice	Hippocampus	Increase in Aβ deposition Microglia activation Astrocyte activation Dystrophic neurites	[5]
		Hippocampus Entorhinal cortex Parietal cortex Striatum Olfactory bulb	Increase in Aβ deposition	[112,113]
	2 months APP23 mice 20–26 months APP23 mice 16 months APP/PS1 mice	Hippocampus	Increase in Aβ deposition Aβ-positive vessels Astrocyte activation Microglial activation Dystrophic neurites	[77]
	20–27 months APP23 mice 20–27 months APP/PS1 mice	Hippocampus	Increase in Aβ deposition	[114]
	18–30 months APP23 mice 18–30 months APP/PS1 mice	Peritoneal cavity	Increase in Aβ deposition	[115]
	84 weeks APP23 mice	Hippocampus	Increase in Aβ deposition	[116]
	25–27 months APP23 mice 20–22 months APP/PS1 mice	Hippocampus	Increase in Aβ deposition	[119]
	2–28.6 months APP23 mice 1.2–22.1 months APP/PS1 mice	Hippocampus	Increase in Aβ deposition	[120]
	24–26 months APP23 mice	Hippocampus	Increase in Aβ deposition	[123]
APP/PS1 mice	2 months APP23 mice 20–26 months APP23 mice 16 months APP/PS1 mice	Hippocampus	Increase in Aβ deposition	[77]
	15–18 months APP/PS1 mice	Sagittal midline Tail veins	Increase in Aβ deposition Vascular amyloid deposition	[122]
5×FAD mice	51 weeks 5×FAD mice 14 weeks 5×FAD mice	Hippocampus	Increase in Aβ deposition	[116]
	21 months APP/PS1 mice	Hippocampus Entorhinal cortex	Increase in Aβ deposition Loss of NeuN	[117]
	10 months 5×FAD mice	Hippocampus	Increase in Aβ deposition Astrocyte activation Microglial activation Demyelination	[118]
Tg2576 mice	18–20 months Tg2576 mice	Hippocampus Peritoneal cavity Thigh Eye	Increase in Aβ deposition Vascular amyloid deposition	[22]
*APP^NL-F/NL-F^* mice	10 months TgCRND8 mice	Right parietal lobe	Increase in Aβ deposition Vascular amyloid deposition	[121]
R1.40 mice	18–30 months APP23 mice 18–30 months APP/PS1 mice	Peritoneal cavity	Increase in Aβ deposition	[115]
	22–25 months APP23 mice	Hippocampus Overlying neocortex	Increase in Aβ deposition	[124]

**Table 3:** Seeding of human brain-derived Aβ seeds in different host animals.

Host Animal	Brain Donor of Aβ Seeds	Injection Site	Induced Pathological Features	Reference
APP23 mice	74- and 85-year-old AD patients	Hippocampus	Increase in Aβ deposition	[77]
	69, 71, and 79-year-old AD patients			[145]
	62-, 81-, and 89-year-old AD patients			[146]
	61-, 64-, 62-, and 85-year-old AD patients	Right parietal lobe	Increase in Aβ deposition Increase in CAA Astrocyte activation Microglial activation	[93]
Tg2576 mice	84-year-old AD patient	Hippocampus Peritoneal cavity Thigh Eye	Increase in Aβ deposition Vascular amyloid deposition	[22]
	81-, 84-, and 91-year-old AD patients	Hippocampus	Increase in Aβ deposition Tau hyperphosphorylation Microglia activation	[125,147,148]
APP/PS1 mice	71–89-year-old AD patients	Hippocampus	Increase in Aβ deposition Induced tau pathology Neurofibrillary tangles Synaptic impairments Memory alteration Neuroinflammation	[149]
	76- and 83-year-old AD patients	Hippocampus	Increase in Aβ deposition Induced tau deposition	[151]
Thy-Tau22 mice	76- and 83-year-old AD patients	Hippocampus	Increase in Aβ deposition Induced tau deposition	[151]
Wistar rats	77- to 87-year-old AD patients	Cerebroventricular	Decreased social memory Loss of volume in the LEC Neuroinflammation Synaptic reorganization	[21]
APP 21 rats	78-year-old AD patients	Hippocampus	Increase in Aβ deposition	[152]
Mouse lemurs	71–89-year-old AD patients	Posterior cingulate Cortex Corpus callosum	Increase in Aβ deposition Vascular amyloid deposition Neurofibrillary tangles Progressive cerebral atrophy Cognitive impairment	[150]
	76- and 83-year-old AD patients	Four different sites surrounding the parietal cortex	Increase in Aβ deposition Induced tau pathology Neuronal loss Cognitive impairments Modifications of neuronal activity	[151]

## Data Availability

No new data were created or analyzed in this study. Data sharing is not applicable to this article.
